# Does a Moderately Warming Climate Compensate for the Negative Effects of UV-B Radiation on Amphibians at High Altitudes? A Test of *Rana kukunoris* Living on the Qinghai–Tibetan Plateau

**DOI:** 10.3390/biology11060838

**Published:** 2022-05-29

**Authors:** Xiaolong Tang, Lu Xi, Zhiyi Niu, Lun Jia, Yucheng Bai, Huihui Wang, Miaojun Ma, Qiang Chen

**Affiliations:** 1 Department of Animal and Biomedical Sciences, School of Life Science, Lanzhou University, No. 222 Tianshui South Road, Lanzhou 730000, China; xil19@lzu.edu.cn (L.X.); niuzhy20@lzu.edu.cn (Z.N.); jial2021@lzu.edu.cn (L.J.); 2 Linxia People’s Hospital, Linxia 731199, China; baiych2009@163.com; 3 Institute of Solid Mechanics, College of Civil Engineering and Mechanics, Lanzhou University, Lanzhou 730000, China; lzu_wanghuihui@lzu.edu.cn; 4 State Key Laboratory of Grassland and Agro-Ecosystems, School of Life Sciences, Lanzhou University, Lanzhou 730000, China; mjma@lzu.edu.cn

**Keywords:** warm temperature, UVB, tadpole, high altitude, development

## Abstract

**Simple Summary:**

Both the warming climate and ultraviolet-B radiation are notable environmental factors affecting tadpole development. However, the phenotypes of tadpoles living at high altitudes may be improved by moderately warming temperatures, reducing or eliminating the negative effects of oxidative damage caused by cool temperatures or strong ultraviolet-B radiation. To verify this hypothesis, *Rana kukunoris* tadpoles, which live at high altitudes, were exposed to ultraviolet-B radiation and ultraviolet-B radiation-free environments at 14 (cool temperature) and 22 °C (warm temperature), respectively. Ultraviolet-B radiation and a warm temperature had opposite influences on several traits of the tadpoles, and the moderate temperature could compensate for or override the negative effects of ultraviolet-B radiation by increasing the tadpoles’ preferred body temperature and critical tolerance temperature, thus enhancing the locomotion ability and thermal sensitivity of their antioxidant systems. The dark skin coloration and aggregation behavior of *R. kukunoris* tadpoles may also be effective strategies for allowing them to resist ultraviolet-B radiation and helping them to better adapt to a warming environment with stronger ultraviolet-B radiation. Thus, a moderate degree of warming may increase the capacity of living organisms to adapt to environmental changes and thus have positive effects on the development of tadpoles living at high altitudes.

**Abstract:**

Both the warming climate and ultraviolet-B radiation (UVBR) are considered to be notable environmental factors affecting amphibian population decline, with particular effects on tadpole development. However, the phenotypes of tadpoles living at high altitudes may be improved by moderately warming temperatures, reducing or eliminating the negative effects of oxidative damage caused by cool temperatures or strong UVBR at high altitudes. To verify this hypothesis, *Rana kukunoris* tadpoles, which live at high altitudes, were used to test the effect of the interaction of temperature and UVBR on their development and antioxidant systems in a fully factorial design. The tadpoles were exposed to UVBR and UVBR-free environments at 14 (cool temperature) and 22 °C (warm temperature), respectively. UVBR and a warm temperature had opposite influences on several traits of the tadpoles, including their survival, developmental rate, individual size, preferred body temperature, thermal tolerance temperature, oxidative damage, and enzymatic and nonenzymatic antioxidant systems. The moderate temperature could compensate for or override the negative effects of UVBR by increasing the tadpoles’ preferred body temperature and critical tolerance temperature, thus enhancing the locomotion ability and thermal sensitivity of their antioxidant systems. Furthermore, the dark skin coloration and aggregation behavior of *R. kukunoris* tadpoles may also be effective strategies for allowing them to resist UVBR and helping them to better adapt to a warming environment with stronger UVBR. Thus, it is possible that a moderate degree of warming may increase the capacity of living organisms to adapt to environmental changes and thus have positive effects on the development of tadpoles living at high altitudes.

## 1. Introduction

In recent decades, human activities have dramatically altered the ecosystem and caused many ecological crises (www.millenniumassessment.org, accessed on 1 December 2021). Among these, a warming climate and increased ultraviolet B radiation (UVBR), associated with stratospheric ozone depletion, are two prominent problems, which may pose serious threats to the survival of many animals and could be the main drivers of global biodiversity loss [1,2]. Many amphibians are sensitive to changes in the environment; in particular, the development process of tadpoles can be significantly affected by warming temperatures and increased UVBR, which could further influence the ecology and population persistence of amphibians in changing environments [3,4,5].

Temperature is one of the most important physical factors of the environment and has profound influences on animal physiological functions, and especially on the thermal biology of ectotherms [6,7]. The thermoregulation and thermal sensitivity of ectotherms, including developing tadpoles, usually reflect changes in environmental temperature [8,9]. The maximum and minimum critical tolerance temperatures (CT max and CT min) reflect the limits of an ectotherm’s performance, and optimal performance usually occurs at a preferred body temperature (T_pre_). Within the critical temperature range, warming temperatures could raise the thermal preferences of individuals living at high altitudes and then positively regulate many physiological and biochemical processes, including tadpoles’ developmental rate, body size, metabolic rate, and many other phenotypes [6,10,11]. In general, tadpoles can reach a higher body temperature through solar heating by situating themselves on a water surface, but this behavior also exposes tadpoles to more ultraviolet radiation (UVR). A certain amount of UVR is necessary for vitamin D synthesis and calcium absorption [12]. However, UVR, especially UVBR, can negatively affect larval growth, even leading to developmental failure and increased mortality [13]. Considering that UVBR and a warm temperature have opposing effects on amphibian thermoregulation, the tadpole may need to make tradeoffs to achieve maximum fitness under the interaction of warming and UVBR.

The interaction of UVBR and temperature on the regulation of reduction–oxidation (redox) homeostasis may alter several traits during tadpole development. Warmer temperatures may induce the formation of more reactive oxygen species (ROS) by increasing the metabolic rate and oxygen consumption in mitochondria [14]. Meanwhile, it will affect the developmental process through the damage of cellular DNA, proteins, and lipids as well as the excessive production of ROS [15]. Both malondialdehyde (MDA) and protein carbonyls are important oxidative damage biomarkers in living organisms. They can disrupt cellular function and, in severe cases, cause mutations and cell death [16]. Nevertheless, organisms can eliminate or scavenge these cellular and molecular damage substances through preventative mechanisms, including UVR-screening compounds (rich in melanin) and an antioxidant system. Darker skin is a basic defense strategy against UVBR [17], and some studies have shown that lower temperatures [18] and UVBR may cause tadpoles to darken in color [19]. The antioxidant systems include both nonenzymatic (glutathione, GSH; oxidized glutathione, GSSG; and total antioxidant capacity, T-AOC) and enzymatic scavengers (catalase, CAT; superoxide dismutase, SOD), respectively. These may remove either oxidant precursors or the oxidants themselves, thereby reducing or preventing cellular oxidative damage [16]. In addition, CPD (cyclobutane pyrimidine dimers) photolyase is a DNA repair enzyme that can repair UVR-induced damage by converting pyrimidine dimers into normal pairs of pyrimidine bases [20]. The balance of intracellular redox homeostasis in species living at high altitudes could be influenced by the extreme environmental factors of the plateau [21], so a warming temperature and strong UVBR could induce more complex changes in the oxidative damage and antioxidant system in animals living at high altitudes.

Globally, amphibians have declined in recent decades, with 41% facing an extinction risk (https://www.iucn.org/, International Union for Conservation of Nature, IUCN, accessed on 1 December 2021). A warming climate and UVR could be important factors affecting amphibian population declines and extinction, especially for species inhabiting high altitudes [22,23,24]. The cool temperatures at high altitudes may exacerbate the negative effects of UVR on the physiological function of individuals [23,25]. However, a moderately warming climate may have positive effects on amphibians, accelerating the metamorphosis of tadpoles and thus improving the survival rate of the population [2,26]. However, empirical evidence for the interaction of a warming climate and UVR on amphibians living at high altitudes, and the relevant mechanisms involved, is still limited. We hypothesized that, for these tadpoles living on plateaus, a warming temperature might improve their thermal fitness and compensate for the negative effects of UVBR damage; if so, the positive effect of a warming temperature may override the negative effect of UVBR, therefore speeding up tadpole development, improving survival rate, and enhancing their capacity to eliminate the oxidative damage caused by UVBR.

To test these hypotheses, we conducted an experiment with a fully factorial design, with UVBR (present/absent) and two constant temperatures (14 and 22 °C) as factors, and analyzed the effects of these two environmental factors on tadpole developmental rate, survival rate, morphology, thermal biology, burst swimming speed, oxidative damage, and antioxidant system. *Rana kukunoris*, an amphibian species living on the Qinghai–Tibetan Plateau, was chosen as a model species in the present study because these two environmental factors are undergoing significant changes in the Qinghai–Tibetan Plateau. Based on satellite data from 1979 to 2008, the UVR was found to increase significantly at 55° S–55° N [27]. Meanwhile, using a statistical regional climate model, researchers have suggested that a 4 °C warming on the Qinghai–Tibetan Plateau will occur from 1950 to 2100, and the warming rate will increase to 0.52 °C per decade from 2020 to 2060 [28,29].

## 2. Materials and Methods

### 2.1. Ethics Statement

This study was carried out with the approval of the Ethics Committee of Animal Experiments at Lanzhou University and based on principles from the China Council on Animal Care. Every effort was made to minimize the numbers used and any suffering experienced by the animals in the experiment.

### 2.2. Experimental Design

The temperature and intensity of UVBR in this study were determined according to the water temperature and UVBR intensity of the sampling ponds. The temperatures of the sampling pools were measured using temperature ibuttons (MD9920, Micronode, Beijing, China) for 7 consecutive days. The temperature of ponds in the field ranged from 11.5 to 21.3 °C, and the mean temperature during activity time (9:00 a.m. to 6:00 p.m.) was 13.55 ± 0.76 °C [30]. The UVB incidence from 1200 to 1400 h on sunny days was measured using a UVB irradiance meter (Tenmars TM-213, Taipei, China). The daily maximum UVB intensity ranged from 5.34 to 7.17 mW/cm^2^, with the mean value being ~6 mW/cm^2^.

According to the temperatures of the sampling ponds, one acclimation temperature was set at 14 °C, which was about the same level as the average temperature in the sampling ponds. The warm temperature group was set at 22 °C, which was based on the maximum daily average water temperature of the field pond and the rate of warming of the Tibetan Plateau every decade [29,30]. Meanwhile, each temperature group was tested with a UVBR-free group and a UVBR group, respectively. The intensity of UVBR was set as 5% of the maximum UVB intensity (~6000 μW/cm^2^), which is 300 μW/cm^2^, to simulate the mean UVR intensity on the Tibetan Plateau.

### 2.3. Animal Collection and Maintenance

*R. kukunoris* is an amphibian species in the *Rana* genus, in the Ranidae family. This species mainly lives in the eastern part of the Qinghai–Tibet Plateau at an altitude of 2000–4200 m. The tadpoles used in the present study were collected from the wetland at Maqu County, Gannan Tibetan Autonomous Prefecture, Gansu Province (33°45′58″ N, 101°44′11″ E, 3490 m a.s.l.), in early May 2021. All these *R. kukunoris* tadpoles (*n* ≈ 500) were randomly collected from different ponds and clutches and subsequently were immediately transported to the Research Station of Alpine Meadow and Wetland Ecosystems of Lanzhou University, Gansu Province, China, Azi Branch Station (33°40′37″ N, 101°52′42″ E, 3530 m a.s.l.), approximately 16 km away from the sampling site.

All tadpoles (Gosner (1960) stage 22–23) were randomly raised in 12 plastic containers (52 × 37 × 31 cm) with a density of 11 individuals per liter, and were fed boiled spinach ad libitum. The water temperature in the container was equal to room temperature (from 8.5 to 12.5 °C) with a 12 h:12 h light–dark cycle. An air pump was used to supply oxygen for 30 min every day to saturate the water with oxygen. All the tadpoles were raised in the lab for about one week and grew to Gosner (1960) stage 25–26. Subsequently, these tadpoles (with a body mass of 0.033 ± 0.001 g and a total length of 14.47 ± 0.16 mm, represented as mean ± SE, *n* = 480) were randomly allocated to four groups, the combinations of which were UVBR free and UVBR (300 μW/cm^2^) with two temperatures (14.0 ± 0.5 and 22.0 ± 0.5 °C). The tadpoles in each group were maintained under the same conditions in three plastic containers (52 × 37 × 31 cm) to maintain a lower density (11 individuals per liter). The water temperature was controlled by aquarium heaters, and the UVBR was generated by a fluorescent light source (Reptisun 10.0, Hong Kong, China) mounted ~23 cm above the containers. The water in the container was changed every three days to maintain water quality. The duration of acclimation was 21 days. Survival was assessed daily, and the development stage of the tadpoles and their morphological characteristics, including the total body length, tail height, and tail length, were determined using ImageJ software (ver. 1.8.0, National Institutes of Health, Bethesda, MD, USA) after photos were taken under a stereoscope (Motic SMZ-168-TL, Hong Kong, China). Soft, absorbent paper was used to soak up the water on the body surface of the tadpoles, and the tadpoles were weighed using an electronic balance (accuracy: 0.001 g). The body length and mass of the tadpoles were recorded at the start and end of the acclimation treatment. Forty tadpoles in each group were euthanized in buffered Tricaine-S (MS-222), frozen in liquid nitrogen, and transferred to our −80 °C refrigerator for measurements of oxidation damage, antioxidant capacity, and gene expression.

### 2.4. Measurement of Thermal Biology

The T_pre_, CTmax, Ctmin, avoidance temperature (AT), and lethal temperature maximum (LTM) were measured after the end of acclimation. The T_pre_ of tadpoles was determined in a temperature gradient chamber (120 × 15 × 30 cm) with a metal tank (61 × 4 × 4 cm) set in the middle of it. Two constant-temperature water baths (set at 70 °C and 0 °C, respectively) were connected to the two ends of the chamber. The water depth of the metal tank was kept at 2 cm so that the tadpole could be exposed to a linear thermal gradient from 7 to 55 °C. Fifty tadpoles were randomly selected from each treatment and divided into 10 groups. For each test, five tadpoles were placed in the metal tank and allowed to swim freely. After acclimating for 30 min, the temperature of each tadpole’s position was represented as the T_pre_ of tadpole and measured using an electronic thermometer (Testo 925, Testo, Lenzkirch, Titisee-Neustadt, Germany). Similarly, eight tadpoles were randomly selected from each treatment for the AT measurement. The tadpoles were placed individually into the metal tank, and the tadpole’s tail was gently touched with a glass rod to make it swim towards the hot end of the tank. The location where the tadpole suddenly turned and began to swim away was recorded, and the water temperature was measured by using an electronic thermometer as the AT of the tadpole.

For the measurement of Ctmax and Ctmin, 10 tadpoles were randomly selected from each group and fasted for 24 h before the experiment. During the test, a tadpole was put into a plastic cup containing 150 mL of water (at room temperature, about 18 °C) for observation. The plastic cup was then put into a water bath, and the water temperature was controlled by heating or cooling at a rate of 1 °C/min. The endpoints of both Ctmax and Ctmin were recorded when the tadpole lost its righting response after being tilted by the gentle touch of a glass rod on its tail [31,32].

At this time, the tadpole was immediately transferred to water at room temperature to allow for recovery. CT max and CT min measurements were nonfatal, and all tadpoles recovered. The temperature of the water when the tadpole lost balance, sank to the bottom of the cup, turned over, had no response to the glass rod touch, and could not be revived within 30 min after the experiment was regarded as the LTM of the tadpole.

### 2.5. Measurement of Burst Swimming Performance

The burst swimming speed was measured using the preferred body temperature apparatus, with some modifications. The temperature in the chamber was controlled by a constant temperature water bath. A small water pump was set in the corner of the chamber to ensure that the water temperature was well distributed. Burst swimming performance was assessed at five temperatures (10, 14, 18, 22, and 26 °C) to generate a thermal dependence curve. The tadpoles were assessed in a metal tank, which was semi submerged in the chamber and filled with water to a depth of 2 cm to prevent vertical movement. Ten tadpoles from each group were randomly selected and placed in the chamber for 10 min to adapt to the test temperature. Startle responses (C-start responses) were elicited by gently touching the tadpole’s tail with a glass rod, and the locomotion of each tadpole was recorded using an iPhone 8 Plus (Apple Inc, Cupertino, CA, USA) at 1080p, 30 fps. Three startle responses were recorded for each tadpole, and the burst swimming speed was analyzed using the Ulead Video Studio 4.0 video editing software (Ulead Systems Inc., Dazzle Multimedia, Fremont, CA, USA). The fastest burst was recorded as the maximum burst performance (Umax).

### 2.6. Measurement of Oxidative Damage and Antioxidant Capacity

To evaluate the effects of temperature and UVBR on the oxidative damage and antioxidant capacity of tadpoles, the concentrations of MDA and carbonyl protein were measured to indicate the degree of oxidative damage, and the activity of CAT and SOD, as well as T-AOC and the GSH:GSSG ratio, were measured to assess the antioxidant capacity of the tadpoles. Whole tadpoles (0.018–0.164 g) were homogenized in 9 volumes of extraction buffer containing a 100 mM potassium phosphate buffer (pH 7.4; KH_2_PO_4_/K_2_PO_4_), 100 mM KCl, and 1 mM ethylene diamine tetra-acetic acid (EDTA). The protein concentration was measured via the Coomassie blue method, using bovine serum albumin as the standard protein. MDA was reacted with thiobarbiturate (TBA) in an acidic boiling water bath to generate a pink complex with a maximum absorption peak at 532 nm. The protein carbonyl content was determined via the DNPH method. The protein carbonyl reacted with 2,4-dinitrophenylhydrazine (DNPH) to form stable dinitrophenyl (DNP) hydrazone adducts, which are red-brown precipitates and can be detected spectrophotometrically at 375 nm. Assays of MDA and protein carbonyl were conducted in duplicate, and the absorbance was read using a microplate reader (SpectraMax M5, Molecular Devices, Downingtown, PA, USA). The concentration of MDA and protein carbonyl in each sample was expressed as nmol/mg protein.

The CAT catalyzed the decomposition of H_2_O_2_ into H_2_O and O_2_ in vivo. The ammonium molybdate quickly stopped this reaction and reacted with the remaining H_2_O_2_ to form a pale yellow complex. After reaction at room temperature for 1 min, the absorbance of the complex was measured using a Microplate Reader (SpectraMax M5, Molecular Devices, Downingtown, PA, USA) at 405 nm. CAT activity was expressed as U/mg protein based on the rate of decrease in the hydrogen peroxide. SOD activity, total antioxidant capacity (T-AOC), and the GSH:GSSG ratio were measured with commercial assay kits (Nanjing Jiancheng Bioengineering Institute, Nanjing, China) following the manufacturer’s instructions. In brief, for SOD activity, a detector of a superoxide radical, water-soluble tetrazolium (WST-1), was generated by xanthine oxidase and hypoxanthine in the presence of SOD [33]. The SOD activity was expressed as U/mg protein. The T-AOC was assessed using the ABTS (2,2′-azinobis (3-ethylbenzothiazoline-6-sulphonic acid)) method described by Re et al. [34]. The T-AOC was expressed as nmol/mg protein based on the rate of the decrease in the hydrogen peroxide. Glutathione and oxidized glutathione were measured using DTNB cyclic reactions. A higher GSH:GSSG ratio usually indicates a stronger H_2_O_2_ scavenging capacity and antioxidant capacity in a living organism.

### 2.7. Quantitative Real-Time PCR

Three tadpoles per treatment were collected after 21 days of exposure and were euthanized by MS-222 for Q-PCR analysis. The total RNA of the tadpoles was extracted using an RNAiso Plus reagent (Taraka, Tokyo, Japan) according to the manufacturer’s protocol. The integrity of RNA was monitored on 1% agarose gel, and the RNA concentration was measured using a NanoDrop 2000 spectrophotometer (Thermo Scientific, Waltham, MA, USA). The RNA samples were reversed into cDNA using a cDNA synthesis kit (Evo M-MLV RT Kit with gDNA Clean for qPCR, Accurate Biology, Changsha, Hunan, China). Expression patterns of CPD photolyase and HSP70 were analyzed through quantitative real-time PCR (qRT-PCR). GAPDH was selected as a reference gene, and the amplified primer sequences were designed using Primer Premier 6 software. The primer sequence of CPD photolyase was F: 5′-TGGCTTCCTCCGTATGTATTGG-3′ and R: 5′-CCGTCCATCCAGTTCATACCG-3′. The primer sequence of HSP70 was F: 5′-GCTCTCGCTCGGAATCGAAA-3′ and R: 5′-GCTGGTTGTCGGAGTAGGTC-3′. Hieff qPCR SYBR Green Master Mix was employed for the performance of qRT-PCR and ABI 7500 Real-Time PCR systems (Applied Biosystems, CA) for determination. The reaction conditions were 95 °C pre-denaturation for 5 min, followed by 40 cycles of 95 °C denaturation for 10 s, 60 °C annealing for 30 s, and 72 °C extensions for 20 s. The 14 °C/UVBR group was used as the control for data analysis through the 2^−ΔΔCt^ method.

### 2.8. Skin Section and Body Coloration Determination

A total of 5 tadpoles per treatment were randomly collected after 21 days of exposure and wholly fixed in a 10% (*v/v*) neutral buffered formalin (NBF) solution. After paraffin embedding, 7 μm thick sections were longitudinally cut and stained with hematoxylin and eosin (H&E) staining using the standard histological protocol. The sections were examined and photographed using a microscope (Shunyu, Nanjing, Jiangsu, China) attached to a digital camera.

Body coloration was determined using the method of Ariel Kruger and Peter J. Morin [35] with some modifications. After 21 days of exposure, 15 tadpoles from each treatment were randomly selected and photographed using an iPhone 8 Plus (F/1.8 aperture, 1/17 s exposure time, ISO 40, no flash). The room light conditions were held constant among individuals. All photos were color-removed and transferred to 8-bit format using Adobe Photoshop CS software (San Jose, CA, USA). The exported photos were then processed using ImageJ software. The elliptical selections tool was used to select the center of the head (about 1/3 of the total head area) or the margin of the tail fin (about 1/5 of the area of the membrane on the dorsal side), and the mean gray value was then measured by adding it into ROI Manager (by default, black is 0, and white is 225).

### 2.9. Statistical Analyses

All the data were tested for normality and homogeneity of variance to meet the assumptions of parametric testing before analysis, and no significant deviations were evident in the data. All the data are represented as means ± SE. Significant differences between groups were analyzed by one-way ANOVA with SPSS 20.0 (IBM SPSS statistics 20.0, SPSS Inc., Chicago, IL, USA), and *p* < 0.05 was considered statistically significant. The result of the homogeneity of variance was shown in the one-way ANOVA analysis, and the LSD post hoc test for data inhomogeneity and Tamhane’s T2 test for data in heterogeneity were used in the post hoc multiple comparisons to detect the differences between every two groups (*p* < 0.05). All graphs were generated using Origin2018 64 Bit (OriginLab, Northampton, MA, USA).

## 3. Results

### 3.1. Development and Survival

**Figure 1:**
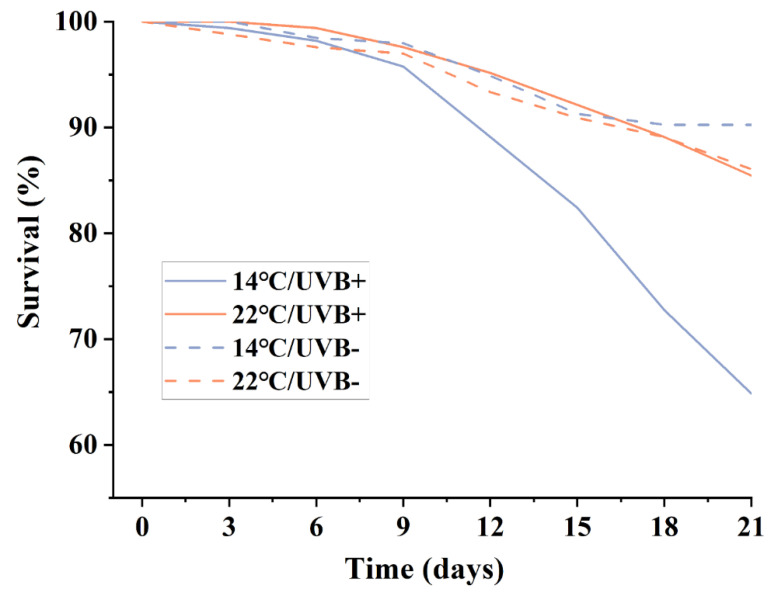
Effect of UVBR (solid lines) and UVBR–free (dashed lines) environments in combination with either a 14 (blue lines) or 22 °C (red lines) temperature on the survival of *Rana kukunoris* tadpoles. Lines show the trend in mortality for each treatment over the 21 day exposure period. The survival of tadpoles in the 14 °C/UVBR group was only 65%, which was lower than that in the other three groups.

After three weeks of exposure, dead tadpoles were found in each group. The survival rate of tadpoles in the 14 °C/UVBR group was only 65%, which was lower than that in the other three groups (Figure 1). The 22 °C condition accelerated the development rate of the tadpoles (Table 1), but the UVBR slowed down the development rate at each temperature treatment. In the 22 °C groups, there was no significant difference in body weight and length between the UVBR and UVBR-free treatments (*F*_3,115_ = 88.093, *p* > 0.05; *F*_3,110_ = 85.224, *p* > 0.01). However, both the body mass and length were lower and shorter in the 14 °C/UVBR group than in the 14 °C/UVBR-free group (*F*_3,115_ = 88.093, *p* < 0.01; *F*_3,110_ = 85.224, *p* < 0.01).

### 3.2. Thermal Biological Characteristics

Both the CTmax and CTmin of the 22 °C groups were higher than those of the 14 °C groups (Table 2). The CTmax of the 14 °C/UVBR group was lower than that of the 14 °C/UVBR-free group (*F*_3,34_ = 15.211, *p* < 0.05), but there was no difference between the two groups at 22 °C (*F*_3,34_ = 15.211, *p* > 0.05). However, the CTmin was significantly increased by UVBR at both temperatures (*F*_3,38_ = 137.952, *p* < 0.01). The thermal tolerance range of the tadpoles in the 22 °C groups was wider than that in the 14 °C groups, but the UVBR induced a narrower thermal tolerance range compared to the UVBR-free treatment.

The T_pre_ of the tadpoles was significantly affected by both a warm temperature and UVBR treatment (Table 2). The T_pre_ in the 22 °C groups was higher than it was in the 14 °C groups (*F*_3,185_ = 129.722, *p* < 0.01). There was no difference in T_pre_ between the UVBR group and the UVBR-free group (*F*_3,185_ = 129.722, *p* > 0.05) at 14 °C. However, the T_pre_ of the tadpoles in the 22 °C/UVBR group was lower than that in the 22 °C/UVBR-free group (*F*_3,185_ = 129.722, *p* < 0.01).

Both the AT and LTM of the tadpoles in the 22 °C groups were higher than in the 14 °C groups (Table 2) (*F*_3,30_ = 30.572, *p* < 0.01; *F*_3,34_ = 20.184, *p* < 0.05). However, the AT in the UVBR group was lower than that in the UVBR-free group (*F*_3,30_ = 30.572, *p* < 0.01), and UVBR did not notably affect the LTM at either of the two temperatures (*F*_3,34_ = 20.184, *p* > 0.05).

### 3.3. Burst Swimming Speed

**Figure 2:**
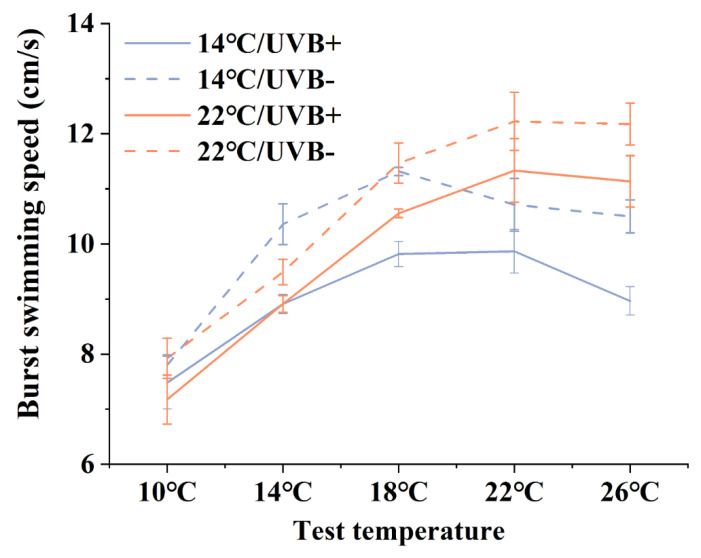
Effect of UVBR (solid lines) and UVBR–free (dashed lines) environments in combination with either a 14 (blue lines) or 22 °C (red lines) temperature on the burst swimming speed in *Rana kukunoris* tadpoles. Lines show the trend in the burst swimming speed for each group at five temperatures (10, 14, 18, 22, and 26 °C), respectively.

The burst swimming speed of the tadpoles follows an inverted U-shaped trend with increasing test temperature (Figure 2). The burst swimming speed was low at 10 °C, and there was no significant difference (*F*_3,30_ = 0.717, *p* > 0.05) among all groups, which suggests that the temperature and UVBR had no effect on the burst swimming speed at low temperatures. Interestingly, when the test temperature was set to the exposure temperatures, the burst swimming speeds of the 14 °C/UVB-free and 22 °C/UVB-free groups were higher (although some comparisons were not statistically different) than those of the other groups, respectively. This suggests that the tadpoles’ burst swimming speed showed a certain thermal adaptability after a period of exposure in the absence of UVBR.

In addition, the UVBR treatment reduced the burst swimming speed of the tadpoles (Figure 2). In the 14 °C groups, the burst swimming speed of the tadpoles in the UVBR group was lower than that in the UVBR-free group at 14, 18, and 26 °C (*F*_3,36_ = 7.299, *p* < 0.01; *F*_3,39_ = 10.184, *p* < 0.01; *F*_3,36_ = 14.636, *p* < 0.01, respectively). In the 22 °C groups, the burst swimming speed of the tadpoles in the UVBR group was lower than that in the UVBR-free group only at 18 °C (*F*_3,39_ = 10.184, *p* < 0.05).

### 3.4. MDA and Protein Carbonyl Determination

**Figure 3:**
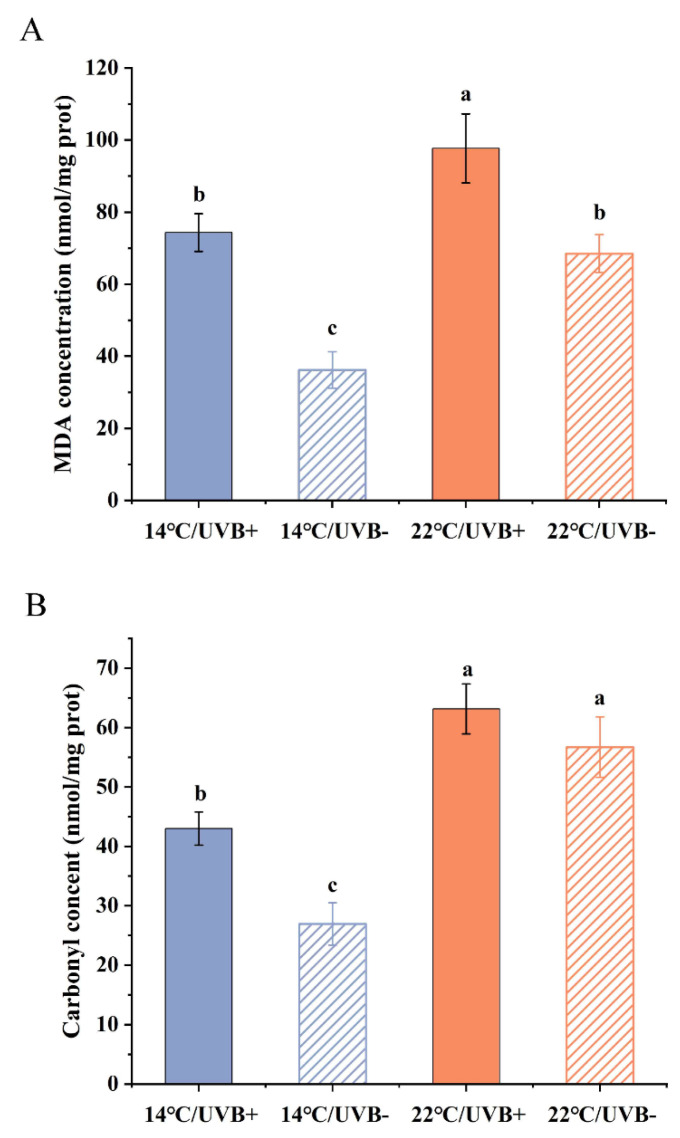
Effect of UVBR (solid bars) and UVBR–free (patterned bars) environments in combination with a 14 (blue bars) or 22 °C (red bars) temperature on the MDA concentration (**A**) and protein carbonyl concentration (**B**) in whole-body *Rana kukunoris* tadpoles. Data are presented as means ± standard error. Lowercase letters above bars denote significant differences between treatments.

The MDA concentration in the UVBR groups was higher than that in the UVBR-free groups at 14 (*F*_3,28_ = 13.071, *p* < 0.01) and 22 °C (*F*_3,28_ = 13.071, *p* < 0.01) (Figure 3A). Furthermore, the UVBR did not affect MDA concentrations in the 14 and 22 °C groups (Figure 3A) (*F*_3,28_ = 13.071, *p* > 0.05), but the MDA concentration in the 14 °C/UVBR-free group was significantly lower than that in the 22 °C/UVBR-free group (Figure 3A) (*F*_3,28_ = 13.071, *p* < 0.01).

The protein carbonyl concentration in the 22 °C groups was higher than that in the 14 °C groups (Figure 3B) (UVBR group: *F*_3,20_ = 13.436, *p* < 0.01, UVBR-free group: *F*_3,20_ = 13.436, *p* < 0.01). Meanwhile, the protein carbonyl concentration in the 14 °C/UVBR group was significantly higher than that of the 14 °C/UVBR-free group (*F*_3,20_ = 13.436, *p* < 0.05), but there was no significant difference in protein carbonyl concentration between the 22 °C groups (Figure 3B) (*F*_3,20_ = 13.436, *p* > 0.05).

### 3.5. Antioxidation System Assay

**Figure 4:**
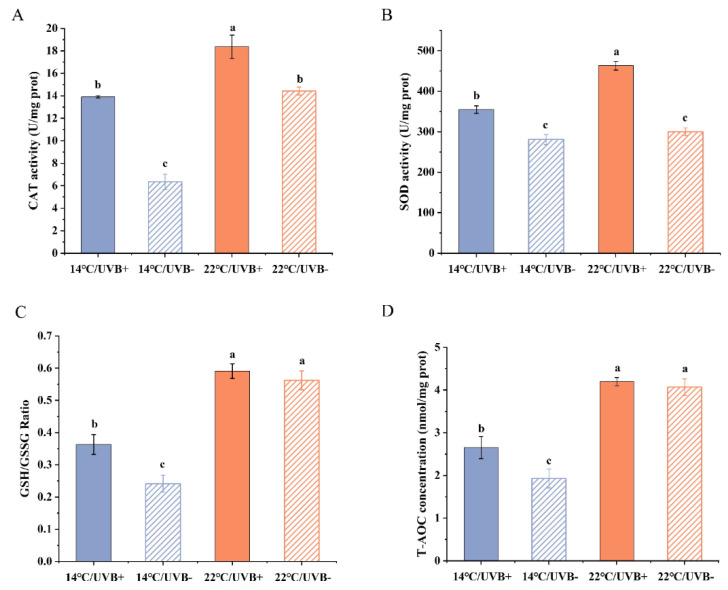
Effect of UVBR (solid bars) and UVBR–free (patterned bars) environments in combination with a 14 (blue bars) or 22 °C (red bars) temperature on the CAT activity (**A**), SOD activity (**B**), GSH/GSSG ratio (**C**), and T-AOC (**D**) in whole-body *Rana kukunoris* tadpoles. Data are presented as means ± standard error. Lowercase letters above bars denote significant differences between treatments.

The CAT activity in the 22 °C groups was higher than that in the 14 °C groups (Figure 4A) (UVBR: *F*_3,22_ = 51.496, *p* < 0.01; UVBR-free: *F*_3,22_ = 51.496, *p* < 0.01). The CAT activity in the UVBR groups was significantly higher than that of the UVBR-free groups at 14 °C (*F*_3,22_ = 51.496, *p* < 0.01) and 22 °C (*F*_3,22_ = 51.496, *p* < 0.01) (Figure 4A).

The SOD activity in the UVBR group was higher than that in the UVBR-free group at both 14 and 22 °C (*F*_3,25_ = 49.7, *p* < 0.01) (Figure 4B). Meanwhile, the SOD activity was only significantly higher in the 22 °C/UVBR group compared to the 14 °C/UVBR group (*F*_3,25_ = 49.7, *p* < 0.01), whereas there was no significant difference between the two UVBR-free groups (*F*_3,25_ = 49.7, *p* > 0.05) (Figure 4B).

The results of total antioxidant capacity (T-AOC) were similar to that of the GSH:GSSG ratio (Figure 4C, D). In both UVBR and UVBR-free groups, the exposure temperatures significantly affected the T-AOC concentration (*F*_3,23_ = 24.644, *p* < 0.01) and the GSH:GSSG ratio (*F*_3,23_ = 30.716, *p* < 0.01). However, the UVBR only improved these two parameters in the 14 °C group (T-AOC concentration: *F*_3,23_ = 24.644, *p* < 0.05; GSH:GSSG ratio: *F*_3,23_ = 30.716, *p* < 0.01), and there is no difference between the UVBR group and UVBR-free group at 22 °C (T-AOC concentration: *F*_3,23_ = 24.644, *p* > 0.05; GSH:GSSG ratio: *F*_3,23_ = 30.716, *p* > 0.05).

### 3.6. Gene Expression of CPD Photolyase and HSP70

**Figure 5:**
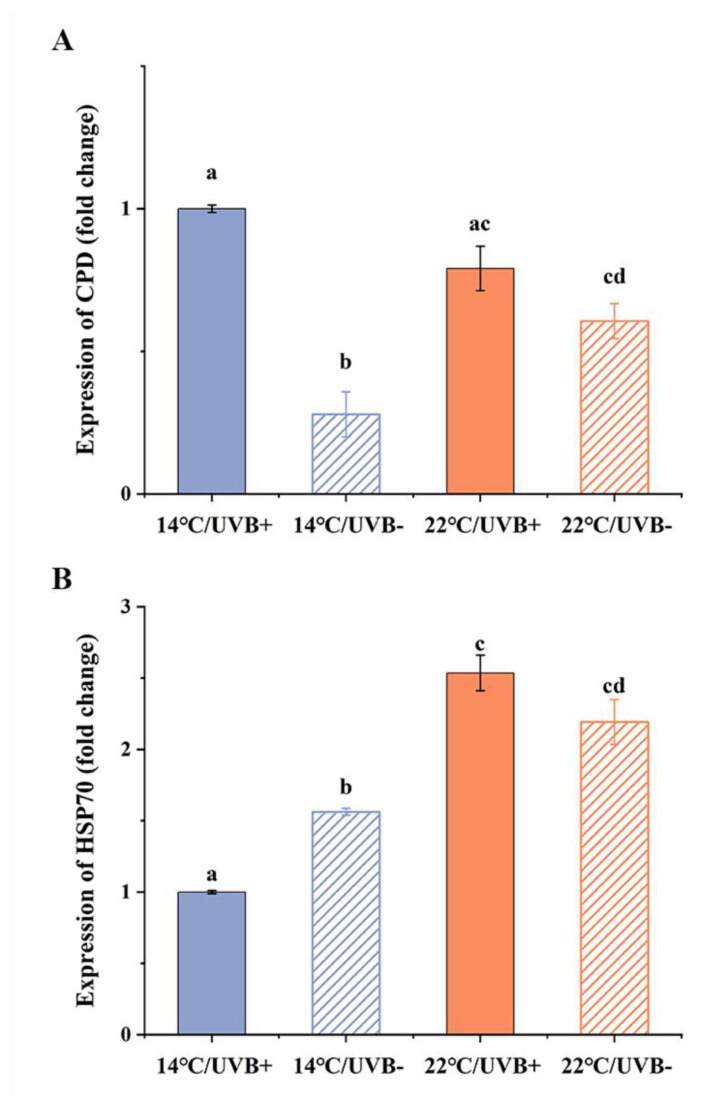
UVBR (solid bars) and UVBR–free (patterned bars) environments in combination with a 14 (blue bars) or 22 °C (red bars) temperature on the expression of CPD photolyase (**A**) and HSP70 (**B**) in *Rana kukunoris* tadpole tail tissue. Data are presented as means ± standard error. Lowercase letters above bars denote significant differences between groups.

The CPD photolyase expression level in the UVBR group was higher than that in the UVBR-free group at 14 °C (*F*_3,15_ = 30.294, *p* < 0.001). Meanwhile, at 14 °C, the CPD photolyase expression level in the UVBR group was significantly higher than that of the UVBR-free group (*F*_3,15_ = 30.294, *p* < 0.05), but there was no difference at 22 °C (*F*_3,15_ = 30.294, *p* > 0.05) (Figure 5A).

The expression level of HSP70 showed that the HSP70 expression level in the 22 °C groups was higher than that in the 14 °C groups (Figure 5B) (UVBR: *F*_3,19_ = 90.654, *p* < 0.001; UVBR-free: *F*_3,19_ = 90.654, *p* < 0.001). Meanwhile, at 14 °C, the HSP70 expression level in the UVBR group was significantly lower than that of the UVBR-free group (*F*_3,19_ = 90.654, *p* < 0.001), but there was no difference at 22 °C (*F*_3,19_ = 90.654, *p* = 0.263) (Figure 5B).

### 3.7. Skin Section and Body Coloration

**Figure 6:**
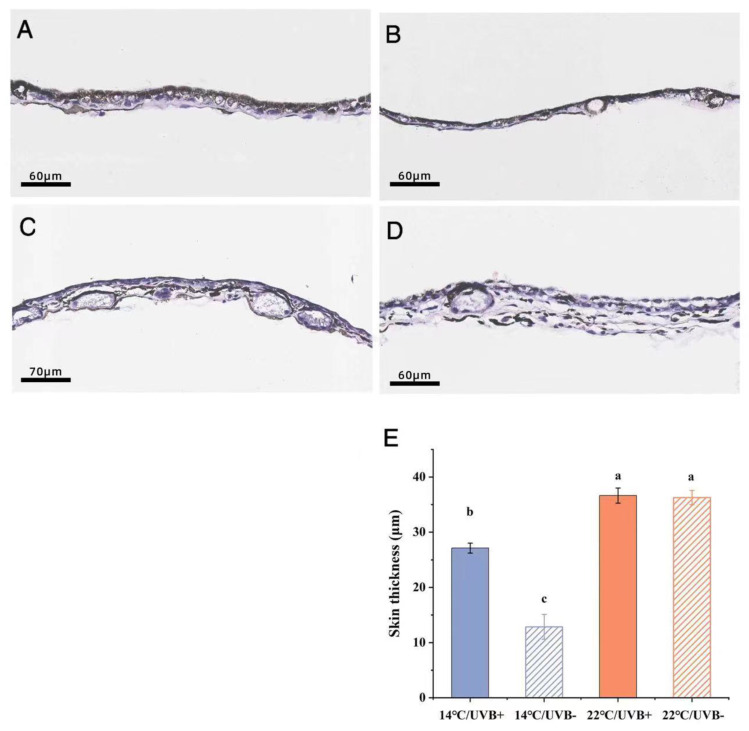
*Rana kukunoris* dorsal skin section. (**A**) Dorsal skin of a tadpole in the 14 °C/UVBR group (Stage G31). (**B**) Dorsal skin of a tadpole in the 14 °C/UVBR–free group (Stage G34). (**C**) Dorsal skin of a tadpole in the 22 °C/UVBR group (Stage G38). (**D**) Dorsal skin of a tadpole in the 22 °C/UVBR–free group (Stage G39). (**E**) Effect of UVBR (solid bars) and UVBR-free (patterned bars) environments in combination with a 14 (blue bars) or 22 °C (red bars) temperature on skin thickness as determined by examining the tadpole sections. Data are presented as means ± standard error. Lowercase letters above bars denote significant differences between groups.

**Figure 7:**
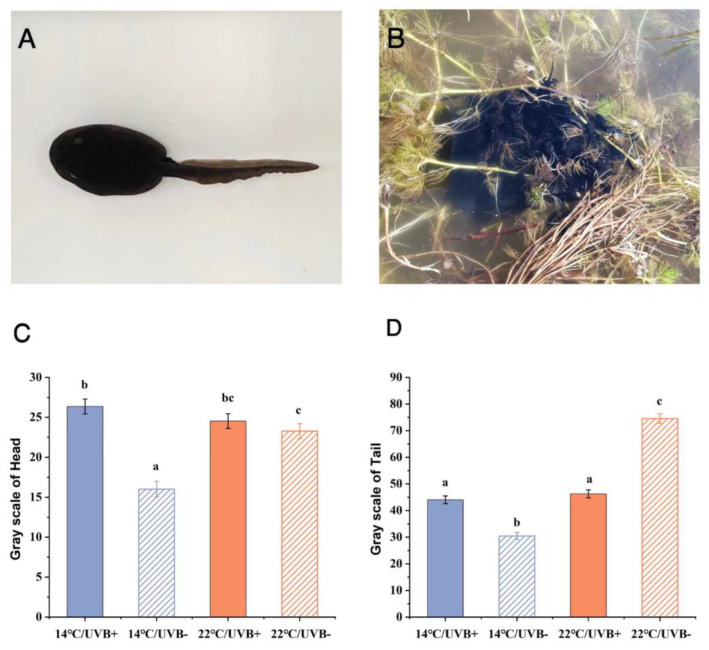
(**A**) *Rana kukunoris* tadpole in Stage G30. (**B**) Aggregation behavior of *Rana kukunoris* tadpoles in a wild pond. (**C**,**D**) Effect of UVBR (solid bars) and UVBR–free (patterned bars) environments in combination with a 14 (blue bars) or 22 °C (red bars) temperature on the grayscale value of the head (**C**) and tail fin (**D**), respectively. Data are presented as means ± standard error. Lowercase letters above bars denote significant differences between groups. The color of the tadpole is expressed by grayscale, with a smaller gray value representing a darker body color. The grayscale value of both the head and the tail fin in the 14 °C/UVBR–free groups was significantly lower than that of the 14 °C/UVBR groups. However, under 22 °C exposure, the grayscale of the tail fin in the UVBR group was significantly lower than that in the UVBR–free group.

Through paraffin section and H&E staining, it was found that the epidermis of the tadpoles in each group contained a large amount of melanin (Figure 6), which could also be the cause of the dark body coloration (Figure 7A). The dorsal skin of the tadpoles in the 14 °C/UVBR group (Stage G31) consisted of a single layer of the epidermis, with highly heterochromatic cell nuclei (Figure 6A). In contrast, the dorsal skin of tadpoles in the 14 °C/UVBR-free group (Stage G34) developed into a two-layer epidermis and included a few scattered glandular-like cells, with characteristics similar to giant cells (Figure 6B) [36]. However, the dorsal skin of tadpoles in the 14 °C/UVBR group was significantly thicker than that in the 14 °C/UVBR-free group (Figure 6E), which could be related to the thicker melanophores and loose skin structure. The dorsal skin of the tadpoles in the 22 °C groups became thicker and developed into a multilayer epidermis, with some granular cells and mucous cells (Figure 6C,D). The UVBR had no significant effect on skin thickness in the 22 °C groups (Figure 6E). However, the skin cells in the UVBR-free group were normal in shape, with round nuclei, while skin cells in the UVBR group were flat, with club-like nuclei. This could be a characteristic of UVBR damage to the skin cells of the tadpoles (Figure 6).

The results of the gray analysis showed that the tadpoles’ heads and tail fins in the 14 °C/UVBR-free group were significantly darker in color than in the 14 °C/UVBR group (Figure 7C,D). However, the color of the tadpoles’ heads was unchanged by UVBR radiation in the 22 °C groups (Figure 7C); only the color of the tail fin in the UVBR group was darker than that in the UVBR-free group (Figure 7D).

## 4. Discussion

In the present study, the interactions of warming temperature and UVBR on the development, thermal biology, locomotion, oxidation damage, and antioxidant system of *R. kukunoris* tadpoles were analyzed at the organismal, molecular, and biochemical levels. According to previous work on amphibian species [4,37], UVBR and a warming temperature can directly influence a tadpole’s thermobiological characteristics and redox hemostasis and further affect survival, developmental rates, and burst swimming speed. These results confirm the hypotheses we proposed and suggest the following conclusions: (1) Increased temperature and UVBR had opposite effects on tadpoles’ thermobiological characteristics, but the positive effects of a moderately warmer temperature on physiological, biochemical, and molecular levels may be greater than the negative effects of UVBR. (2) In response to a strong UVBR at a high altitude, *R. kukunoris* tadpoles can reduce oxidation damage through different strategies, including darker skin and an enhanced antioxidation system.

### 4.1. Thermal Biology

Tadpoles regulate their body temperature by exchanging heat with their habitat environment and, consequently, are particularly vulnerable to ambient temperature variation [38]. Studies both in the wild and in laboratories have shown that the thermal fitness of tadpoles can increase with exposure to a moderately warmer temperature [2,39]. Such a conserved thermal response could be positively correlated with the expression of heat shock proteins (Hsps) [40]. Hsp expression can be affected by heat, cold, or UVBR, and these stresses can be countered mainly by molecular chaperone activity [41]. The induction of these stress response genes has been correlated with enhanced thermoresistance [42,43]. In the present study, the T_pre_, CTmax, CTmin, AT, and LTM of *R. kukunoris* tadpoles increased more or less after exposure to a 22 °C temperature (Table 2), which showed the same trend as that of the expression of Hsp70. These results suggest that *R. kukunoris* tadpoles living at high altitudes can quickly acclimate to moderately warming temperatures at a molecular level. This conservative response to temperature change could be more effective for ectotherms that live in cold-temperature habitats [44] and may make it possible for *R. kukunoris* specimens to enhance their thermoregulation ability to cope with warming climates in the future.

UVBR has also been shown to affect the expression of Hsps and plays a direct or indirect role in thermal optima and resistance [45]. In the present study, UVBR had reverse effects on the thermal optima and resistance of *R. kukunoris* tadpoles, including the narrowing of the critical temperature range in parallel with a lower AT at both temperatures, as well as a lower T_pre_ in the 22 °C groups. Meanwhile, the expression of Hsp70 in the 14 °C/UVBR group was lower than that in the 14 °C/UVBR-free group, but there was no change between the 22 °C/UVBR group and the 22 °C/UVBR-free group. Therefore, these results indicate that the interaction of UVBR and a cold temperature may affect the thermoresistance and preference of tadpoles by downregulating the Hsp70 expression level, or negatively affecting the synthesis or folding of HSP proteins [46]. In reverse, moderate warming could override the negative effect of UVBR on Hsp70 expression and help *R. kukunoris* tadpoles to survive in extreme environmental conditions on plateaus. However, under the interaction of multiple environmental factors, the effect of Hsps on tadpole thermoregulation and thermal resistance is unclear. This is an important unanswered question that will drive future research.

### 4.2. Development, Survival, and Locomotion

The effects of a warming temperature and UVBR on thermal fitness can further change the physiology and behavior of tadpoles, especially their survival and developmental rate. Numerous studies have shown that, in general, warm temperatures can accelerate tadpole development and improve survival, while UVBR has adverse effects [22,47]. However, the interactions of these two environmental factors on the developmental rate and survival have been reported to differ greatly among diverse species [22,48]. A study on *Limnodynastes peronii* showed that the developmental rate of tadpoles was significantly accelerated when the temperature was increased from 15 to 26 °C, but it was not affected by different levels of UVR [4]. Similar results have also been reported for *Anaxyrus boreas* and *Pseudacris regilla* [49]. In contrast, studies on *Platyplectrum ornatum*, *Litoria verreauxii*, and *Crinia signifera*, as well as our present study on *R. kukunoris* tadpoles, indicate that a cool temperature can enhance the negative effects of UVBR, as the tadpoles showed a significantly reduced development rate and greater mortality [50,51]. The apparent thermal dependence of UVBR effects may be due to the reduced UVR defense ability at lower temperatures [37]. This also indirectly explains why UVBR is an important cause of the current decline in amphibian populations at high altitudes with cooler temperatures [52,53]. Conversely, a warming climate could help tadpoles to mitigate the negative effects of UVBR on their growth and survival. It may be that tadpoles living at high latitudes or high altitudes can develop rapidly and metamorphose before their habitat disappears, which is related to accelerating evaporation rates caused by warming temperatures [49].

Temperature and UVBR also had notable effects on tadpole swimming performance. The *R. kukunoris* tadpoles showed faster burst speeds at their exposure temperatures, which indicates that the locomotion of tadpoles can be well matched to their experienced thermal conditions [54]. This is mainly due to changes in temperature affecting the activities of receptors and enzymes that maintain the normal function of the tadpole’s tail muscles, including excitation–contraction coupling control and calcium cycling [55]. These thermally adaptive changes in enzyme activity, in parallel with the plasticity of locomotion matching to their thermal environment, could help tadpoles to better adapt to a future warming climate [56]. On the other hand, similarly to the negative effects of UVBR on development and thermal fitness, UVBR also reduced the burst swimming speed of *R. kukunoris* tadpoles. The negative effects of UVBR on locomotor performance and muscle contraction function could mainly be due to ROS that damage sarcolemma and some locomotion-related proteins, such as myosin ATPase and sarcoplasmic reticulum Ca^2+^-ATPase [57]. In addition, UVBR may reduce the muscular activity of tadpoles and thus minimize intrinsic ROS production [58] but not affect the ATP production of mitochondria [59]. Moreover, in the present study, although UVBR had negative influences on locomotion, the burst swimming speed of *R. kukunoris* tadpoles in the 22 °C/UVBR group was faster than that of the 14 °C/UVBR group. This suggests that moderate warming can override the negative effects of UVBR and enhance the locomotion of *R. kukunoris* tadpoles, and thus may benefit their predation, behavioral thermoregulation, and metamorphosis speed.

### 4.3. The Oxidation and Antioxidation Systems

Numerous studies have shown that increased temperature and UVBR can induce more ROS production through different pathways and mechanisms [15,60]. For *R. kukunoris* tadpoles living at high altitudes, both a warm temperature and UVBR will exacerbate oxidative damage, but the antioxidant scavenging system may also change with the environmental variation. The results regarding the oxidative damage and antioxidant system in *R. kukunoris* tadpoles confirmed our hypothesis and indicated that, when the exposure temperature increased from 14 to 22 °C, the UVBR-induced oxidative damage decreased. Meanwhile, there was no effect of UVBR on the carbonyl protein formation, T-AOC activity, and GSH:GSSG ratio in the 22 °C groups. The *R. kukunoris* tadpoles counteracted the additional metabolic ROS production by utilizing the thermal sensitivity of the antioxidant system, especially for the activity of the antioxidant enzyme, without increasing the abundance of nonenzymatic antioxidants [13,61]. This temperature-dependent regulatory strategy could be even more important for species that live at higher altitudes, which need to withstand cooler and greater fluctuations in ambient temperatures. These results also suggest that the effect of a warming temperature will compensate for the negative effects of UVBR by altering the redox balance of the organism while influencing the survival, development, locomotion, and other phenotypes of the tadpoles [13,14].

The regulation of CPD photolyase gene expression is usually determined by the accumulation of DNA damage in the epithelial cells [45]. Interestingly, the CPD photolyase of *R. kukunoris* tadpoles was only highly expressed (2–3-fold) in the 14 °C/UVBR group. This result suggests that a cool temperature can enhance the accumulation of UVBR-related DNA damage, which could also indirectly explain the lower survival in the 14 °C/UVBR group. Consistent with earlier work on *L. peronii* [4], there was no significant effect of UVBR on CPD photolyase expression in *R. kukunoris* tadpoles in the warm-temperature treatments. This indicated that the expression of CPD photolyase after exposure to the warm temperature was sufficient to cope with the DNA damage induced by UVBR. However, a separate study, also on *L. peronii*, showed that, when the tadpoles were acutely exposed to either a higher temperature or UVBR, the expression of CPD photolyase was upregulated [25]. These results suggest that the expression of CPD photolyase may depend on not only the level of temperature but also the duration of temperature and UVBR exposure. In addition, as *R. kukunoris* is a species living at high altitude, some UVBR-screening-related protection strategies, including the skin color and skin structure of tadpoles, may reduce the UVBR-caused damage to the living organism and therefore influence the expression of CPD photolyase in response to UVBR.

The skin of tadpoles generally thickens during the developmental and metamorphic process, developing from a simple monolayer of epidermal cells into a multilayer cellular structure consisting of epidermis and dermis [62]. The warming effect accelerated the developmental rate of *R. kukunoris* tadpoles and increased skin thickness. A thicker skin and secretions from granular cells and mucous cells can effectively protect tadpoles from UVBR [17]. In addition, the presence of glandular giant cells may be associated with the gregarious behavior of tadpoles [62]. *R. kukunoris* tadpoles usually congregate in large numbers at the surface of water in the wild (Figure 7B). The tadpoles can obtain a greater surface area by huddling together to increase the intensity of solar radiation absorbed from the sun [63]. Although this behavior puts tadpoles at risk of exposure to increased UVBR, given their long-term evolution and adaptation at high altitudes, the uniformly blackish skin color of *R. kukunoris* tadpoles helps them to absorb heat from the sun’s radiation and ensure their protection from UVBR damage [64].

Dark skin coloration is the first barrier against UVBR effects for tadpoles [65]. In general, increased temperature or UVBR will significantly improve melanin synthesis [66,67]. However, the color of the dorsal and tail fins of *R. kukunoris* tadpoles in the 14 °C/UVBR-free group was darker than that in the other three groups. This may have been caused by the negative effect of UVBR on the cells of the skin, affecting the synthesis of melanin. It is also possible that cooler temperature conditions restricted the tadpoles’ energy intake, and the limited energy may have been more allocated to other key functions, including increased photo-enzymatic production and cell repair [68] or in maintaining the normal functional response of the immune system [69]. As a result, in the 14 °C/UVBR group, the melanin synthesis of the tadpoles was reduced, and the body color was relatively light.

## 5. Conclusions

Our results indicate that UVBR can negatively affect several phenotypic characteristics of tadpoles during development, but a moderate increase in exposure temperature revealed a compensation effect by changing the thermal fitness traits and thermal sensitivity of the living organism. The T_pre_, CT max, and oxidant scavenging capacity were enhanced and therefore eliminated the negative effects of UVBR. These results have implications for our understanding of what a warming temperature and UVBR may contribute to tadpoles’ developmental process, and suggest that a moderately warming temperature may have a positive impact on amphibians that live at high altitudes, which may positively affect the decline of the amphibian population in plateau environments. In the future, experiments conducted in semi wild enclosures or interactive effect analyses of fluctuating temperatures and UVBR may provide further evidence that supports this hypothesis.

## Figures and Tables

**Table 1:** Variation in the developmental rate, body weight, and body length of *R. kukunoris* tadpoles. The developmental stages are expressed in Gosner modes (1960). The body mass and total body length are represented as mean ± SE. Lowercase letters above bars denote significant differences between groups.

Group	Developmental Stage	Body Mass (g)	Total Body Length (mm)	Tail Length (mm)	Tail Height (mm)
Initial period	26 (25–27)	0.033 ± 0.001	14.401 ± 0.158	8.063 ± 0.088	2.620 ± 0.045
14 °C/UVBR	31 (30–32) ^d^	0.153 ± 0.004 ^c^	23.458 ± 0.273 ^d^	13.398 ± 0.174 ^c^	4.388 ± 0.178 ^b^
14 °C/UVBR-free	34 (32–34) ^c^	0.195 ± 0.005 ^b^	24.909 ± 0.304 ^c^	13.869 ± 0.150 ^c^	4.65 ± 0.084 ^a^
22 °C/UVBR	38 (37–39) ^b^	0.232 ± 0.003 ^a^	27.899 ± 0.251 ^b^	16.023 ± 0.184 ^b^	4.74 ± 0.065 ^a^
22 °C/UVB-free	39 (38–41) ^a^	0.235 ± 0.004 ^a^	28.817 ± 0.214 ^a^	16.917 ± 0.176 ^a^	4.783 ± 0.052 ^a^

**Table 2:** Variation in the critical thermal maximum, critical thermal minimum, thermal tolerance range, preferred temperature, avoidance temperature, and lethal thermal maximum of *R. kukunoris* tadpoles. All data are represented in mean ± SE. CTmax: critical temperature maximum; CTmin: critical temperature minimum; ΔCT: critical temperature range; Tpre: preferred body temperature; AT: avoid temperature; LTM: lethal temperature maximum. Lowercase letters denote significant differences between groups.

Group	CT_max_ (°C)	CT_min_ (°C)	ΔCT (°C)	T_pre_ (°C)	AT (°C)	LTM (°C)
14 °C/UVBR	30.51 ± 0.80 ^c^	4.28 ± 0.17 ^c^	26.24	18.14 ± 0.30 ^c^	26.66 ± 0.18 ^c^	38.35 ± 0.09 ^c^
14 °C/UVBR-free	31.88 ± 0.74 ^b^	3.08 ± 0.12 ^d^	28.80	18.12 ± 0.27 ^c^	28.80 ± 0.28 ^b^	38.72 ± 0.13 ^c^
22 °C/UVBR	36.02 ± 0.62 ^a^	8.69 ± 0.20 ^a^	27.33	23.08 ± 0.45 ^b^	28.81 ± 0.27 ^b^	40.60 ± 0.27 ^a^
22 °C/UVB-free	36.09 ± 0.65 ^a^	7.03 ± 0.27 ^b^	29.06	25.06 ± 0.19 ^a^	30.43 ± 0.35 ^a^	39.98 ± 0.24 ^b^

## Data Availability

The original contributions presented in the study are included in the article. Further inquiries can be directed to the corresponding authors.
